# Transformations between rotational and translational invariants formulated in reciprocal spaces

**DOI:** 10.1016/j.yjsbx.2023.100089

**Published:** 2023-06-02

**Authors:** Philip R. Baldwin

**Affiliations:** Baylor College of Medicine, One Baylor Plaza, Houston, TX 77030, USA

**Keywords:** Bispectrum, Wilson statistics, Shape analysis

## Abstract

Correlation functions play an important role in the theoretical underpinnings of many disparate areas of the physical sciences: in particular, scattering theory. More recently, they have become useful in the classification of objects in areas such as computer vision and our area of cryoEM. Our primary classification scheme in the cryoEM image processing system, EMAN2, is now based on third order invariants formulated in Fourier space. This allows a factor of 8 speed up in the two classification procedures inherent in our software pipeline, because it allows for classification without the need for computationally costly alignment procedures.

In this work, we address several formal and practical aspects of such multispectral invariants. We show that we can formulate such invariants in the representation in which the original signal is most compact. We explicitly construct transformations between invariants in different orientations for arbitrary order of correlation functions and dimension. We demonstrate that third order invariants distinguish 2D mirrored patterns (unlike the radial power spectrum), which is a fundamental aspects of its classification efficacy. We show the limitations of 3rd order invariants also, by giving an example of a wide family of patterns with identical (vanishing) set of 3rd order invariants. For sufficiently rich patterns, the third order invariants should distinguish typical images, textures and patterns.

## Graphical abstract

**Figure:**
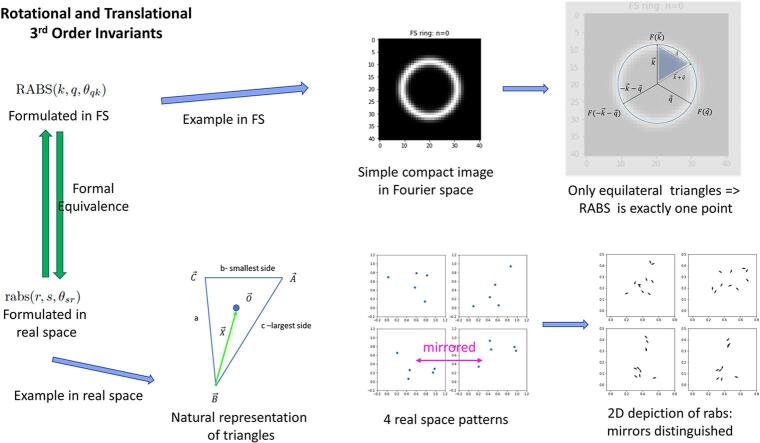

## 1 Introduction

Correlation functions play a crucial role in the formulation of many ideas in random walks, field theory, statistical mechanics and structural biology. In the realm of classification purposes, they have found uses in EEG (Wang et al., 2015), characterization of seismic waves (Hocke and Kümpfer, 2008), MRI (Friedlinger et al., 1999), and generally as shape descriptors (Kakarala, 2012). Indeed bispectra have found ample use in astronomy in classification of angular patterns in cosmic microwave background (CMB) (Scoccimarro, Dec 2000, Huang and Vernizzi, Mar 2013, Fergusson and Shellard, Aug 2009) and more recently trispectra (Lee and Dvorkin, May 2020, Bertolini et al., Jun 2016). In speckle interferometry, they are used to find model parameters in star systems by averaging bispectra (Hofmann et al., 2019). In quantum information science, nonGaussianinity, as measured by multispectra is also starting to gain a foothold (Ramon, s2019).

In cryoEM, it was an early goal to use image invariants built from correlation functions like power spectra and double auto correlation functions to characterize misaligned images, (Frank, 2006) since invariants do not depend on the arbitrary choice of origin and only on the image content. However, the early sets of invariants were deficient in that they cannot, at best, capture more than 1/4 the amount of the original information in an image. Early attempts to solve this shortcoming and use multispectra did not gain a strong foothold (Marabini and Carazo, 1996). As computational power increases, multispectra are being studied with the intent of creating models directly from micrographs (Lan et al., 2022). Among these topics, our work lies closest to the CMB literature, in that this literature typically focusses attention to specific patterns of wavevectors (Lee and Dvorkin, May 2020).

The group of motions representing rotations and translations is generally termed the Euclidean group, or, more simply, the motion group. The associated invariants we will therefore term “motion invariants”. We seek motion invariants so as to do away with the very costly step of aligning images in order to classify them. Motion invariants involve constructing correlation functions and performing a final rotational average, and are usually formulated in Fourier space, because they generally are typically faster to evaluate in this representation. Additionally Fourier space is often a more natural variable due to the fact that SNR is more naturally formulated there. Our present work is the first place, to our knowledge, that has shown the efficacy of a real space formulation of higher order correlation functions, and a better set of variables to describe them, based on making the parametrization more symmetric. Formulation in real space, although possibly slow, avoids aliasing artifacts and can be tailored for specific patterns that are expected to show correlations.

A question arises, then, if such rotational averages (a sort of projection) are taken in both real and reciprocal spaces, whether there is always a formula by which one can transform from one set of these resultant correlation functions to another, meaning that there is no larger set of invariants one can form by developing invariants in both spaces. The theoretical development lies ultimately in the language of irreducible representations (Isaacs, 1976), where the invariants play the role of group characters. Indeed, there is much akin in the character tables of irreducible representations of symmetries, where solving for the characters (Wilson et al., 1955) is necessary to predict the strength of spectroscopic lines in Raman and vibrational spectroscopy. Here one must account for the possible motions of a molecule constrained by the symmetries, which bears some similarities to the formalism of our work.

**Fig. 1:**
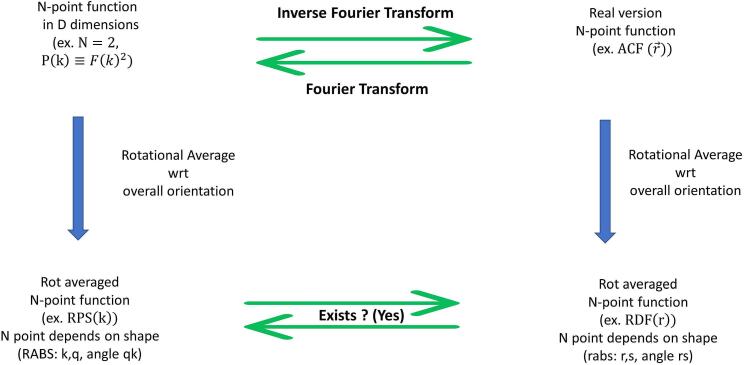
N Point Correlation Functions and their projections into symmetric subspaces. In this manuscript, we show that after a rotational averaging (downward arrows), that one may always transform back and forth between the resulting translational and rotational invariants for all n-point functions and in all dimensions. We explicitly evaluate the kernels in D = 2 for 3 point functions.

The situation is summarized in Fig. 1, where the projection discussed there, is a rotational average of the correlation function. As a simple, and well known (Hua, 2005, Takeshi et al., 2012) example of relating invariants in reciprocal spaces, consider the formula relating radial power spectrum (RPS) and radial distribution function (RDF) of 3D space:

(1.1) $$\mathrm{RPS}\left(k\right)=4\pi \int_{0}^{\infty }t^{2}\;dt\;\frac{\sin\mathrm{kt}}{\mathrm{kt}}\;\;\mathrm{RDF}\left(t\right)\;.$$

The function RPS(k), formulated from the rotational averages of the power of the Fourier transform, is directly related to RDF(t) formulated from the rotational average of the auto correlation function, with a similar looking inverse relationship.

One goal in this monograph is to provide equations for other n-point functions and other dimensions, *D*. In principal, we could write down general formulas for arbitrary *n* and *D* with the approach here, but it is more practical to give expressions for D=2,3,4 and n=2,3,4. We give some non-trivial particular cases in great detail for n=3 (bispectrum) and D=2. That such transformations exist, depends on the fact that the (Fourier) transforms in question are axial, as discussed at length in the main body: because the transform is expressed as a function of the inner product of real and reciprocal variables, symmetries are identical in reciprocal spaces.

One is therefore at liberty to formulate motion invariants in the space, which is most convenient: typically the space in which the original signal is most compact. As a calculational exercise, we give examples of dense 2D patterns (that have support on a circle in Fourier space) that have highly degenerate 3rd order patterns, taking on measure at either a single point or even completely vanishing. Conversely, for highly singular real space signals, we give real space expressions for the third order correlation function, which is equivalent to encoding all of the possible triangles in the original signal. The natural coordinates in this three dimensional representation are derived to be an alternative and more symmetric form than Bookstein coordinates (Dryden and Mardia, 2016) for triangles, which is a staple of the study of statistical shape analysis. Mirrored triangles may be found by flipping the sign of a phase angle in this triangle representation and are therefore distinguished. Together the above facts show that third order invariants distinguish mirrors, but generally cannot completely recapitulate the image.

In order to perform the transformations between reciprocal spaces, we ultimately need to understand the parametrizations of rigid polytopes. It is necessary to determine how many variables are involved in the expression of the projected (rotationally averaged) correlation function, and how many variables are involved in the convolution to transform the invariants, which we elaborate in the table below. To understand the nuances here, consider that it takes 5 coordinates (not 6, which is the number of pairs of vertices, and is correct for D≥3) to parametrize a 4 vertex polytope in 2D (n=4,D=2). From the table, the internal structure of the polytope gives the dimension of the final invariant, whereas the reorientation gives the order of the integration needed to give the final rotational invariant (Table 1).

**Table 1:** Decomposition of the degrees of freedom of a rigid polytope of *n* vertices in *D* dimension. Understanding this decomposition is necessary for writing down the kernel for transforming invariants between reciprocal spaces. For a triangle (n=3), for example, in D=2, this corresponds to the middle case, which we have called “Saturated”. The internal structure is essentially determined by the n(n-1)/2=3 side lengths of the triangle, and the reorientation is governed by the D(D-1)/2=1 orthogonal group in that dimension. Given *D*, smaller or larger *n* will create the unsaturated or oversaturated cases. In the prior case, the internal structure is governed by n(n-1)/2 (bold face in the table), and in the latter case, the reorientation (bold face) is governed by orthogonal group as before. However both expressions are only simultaneously true for the saturated case. In each case, the DOF (*nD*) is equal to the sum of the last three columns.

The manuscript is organized as follows. In Section 2, we cover the power spectrum in great detail for dimensions 2, 3, and 4. In Section 3, we handle the 3 point function only for D = 2, but the same sort of analysis can be developed in other dimensions and other number of points.

Gaussian waveforms wind up being extremely useful simplification of signals in many areas of signal analysis. Although, strictly speaking, they do not satisfy general criteria to form good basis functions, their implementation has been of the utmost of usefulness in disparate areas of signal processing, including structural biology (Wilson, 1949), electrical engineering and quantum optics (Grynberg et al., 2010). For the purposes of cryoEM, Gaussian waveforms satisfy many remarkable properties: such as 1. convolutions of Gaussians remain Gaussian, and 2. projections of Gaussians are Gaussian. In Section 4, we reformulate the equations of Section 3 in terms of a Gaussian decomposition of the original signal. As suggested above, this allows us to very easily see that third order invariants distinguish mirrors, and it is easy to give closed form solution for the motion invariants. For highly singular signals of identical strengths, the invariants are the enumeration of the different types of polytopes that one can find in the pattern of the signal. This elucidates most clearly the meaning of the correlation functions. However, when specific patterns of sampling points are desired, then it is much easier to formulate in real space.

Section 5 is our results section, where we show examples of 3 point functions formulated in both Fourier and real spaces, as well as a tentative discussion of some part of the 4 point correlations. Specifically, we show that mirrored objects can be distinguished by the 3 point functions in 2D, and that chiral objects can be distinguished by 4 point functions (but not 3pt functions) in 3D. Section 6 is a discussion and conclusion. In practice, motion invariants built from correlation functions are easier to interpret from their real space versions, but typically faster to evaluate from Fourier versions. The reason is that the correlation functions already involve a convolution over the entire space, which is easily handled in the Fourier transform domain. On the contrary, if a definite set of sampling points is known to be salient, it is numerically easiest to formulate the problem in real space directly, which avoids a full evaluation of a multispectrum.

## 2 Radial Power Spectrum (RPS) and Radial Distribution Function (RDF) in D = 2,3 and 4

The simplest set of rotational and translational invariants are due to the two point function, RPS and RDF which form a key part of the theory of scattering in a wide array of systems (Hua, 2005, Takeshi et al., 2012). They form an incomplete description of a pattern, because they are based on the squared modulus of the Fourier transform, so that all the phase information is lost. In this section, we show the equivalence of the rotational invariants formed by the two point function: RPS, which is formulated in Fourier space, and RDF in real space. The construction for the transcription for the higher order correlation functions take place with a similar mechanism. More mathematical details are given in Appendix A. Consider a real 2D signal, *f*, and its Fourier transform, *F*, as well as its squared modulus:

(2.1) $$F(\overset{\to }{k})\equiv \int_{\overset{\to }{r}}f(\overset{\to }{r})\;e^{i\overset{\to }{k}\cdot \overset{\to }{r}}$$

(2.2) $$|F(\overset{\to }{k}){|}^{2}=\int_{\overset{\to }{r},\overset{\to }{s}}f(\overset{\to }{r})f(\overset{\to }{s})e^{i\overset{\to }{k}\cdot (\overset{\to }{r}-\overset{\to }{s})}\text{.}$$

Then RPS is defined as

(2.3) RPS(k)≡1SD∫k^|F(k→)|2,

(2.4) =SD∫0∞dttD-1KerktRDFt,Kerkt≡1SD∫k^eik→·t→,

with RDF being defined as

(2.5) RDF(t)≡1SD∫s→,t^f(s→)f(s→+t→),

and $S_{D}$ is the volume of the d-dimensional ball.

For D=3, the expressions become

(2.6) $${\mathrm{Ker}}_{D=3}(x)=\frac{\sin x}{x},$$

(2.7) $$\mathrm{RPS}(k)=4\pi \int_{0}^{\infty }t^{2}\;dt\;\frac{\sin\mathrm{kt}}{\mathrm{kt}}\;\;\mathrm{RDF}(t),$$

(2.8) $$\mathrm{RDF}(t)=\frac{1}{2\pi^{2}}\int_{0}^{\infty }k^{2}\;dk\;\frac{\sin\mathrm{kt}}{\mathrm{kt}}\;\;\mathrm{RPS}(k)\text{.}$$

The expressions for D=2,4 are given in the appendix A, and are similar in spirit. It is not so much the derivation or the formula that we want to stress as that the transformation between invariants is an involution: they contain equivalent information. More details of the derivation are given in the appendix. One is at liberty to create the set of invariants in the space that is more convenient. Reasons to choose real space invariants is that i) they can be evaluated without aliasing, ii) it is easier to demonstrate the mirroring properties for third order invariants, which we shall see in the next two sections.

## 3 RABS: Fourier space representation and transformation to real space

In the last section, we showed the equivalence of the two point correlations, and in this section we demostrate the analogous constructions for the 3PCF (three point correlation function: the term used in cosmology), which we call RABS (rotationally averaged bispectrum). Again, the issue is that one can get invariants in both spaces, presumably in the space that is more convenient. The RABS is easier to develop computationally in Fourier space generally, however we find it easiest to describe intuitively the meaning of the rotationally averaged three point function as the resonance of the original signal with triangles of a given shape. (For this line of thinking, see (Shmahalo, 2019) based on (Baumann et al., 2022)).

To proceed, we formulate the 3PCF function in Fourier Space (where *B* means bispectrum):

(3.1) $$B(\overset{\to }{k},\overset{\to }{q})\equiv F(\overset{\to }{k})F(\overset{\to }{q})F(-\overset{\to }{k}-\overset{\to }{q}),$$

(3.2) $$=\int_{\overset{\to }{r},\overset{\to }{s},\overset{\to }{t}}f(\overset{\to }{t}+\overset{\to }{r})f(\overset{\to }{t}+\overset{\to }{s})f(\overset{\to }{t})e^{i\overset{\to }{k}\cdot \overset{\to }{r}}e^{i\overset{\to }{q}\cdot \overset{\to }{s}},$$

(3.3) $$=\int_{\overset{\to }{r},\overset{\to }{s}}e^{i\overset{\to }{k}\cdot \overset{\to }{r}}e^{i\overset{\to }{q}\cdot \overset{\to }{s}}\;\;b(\overset{\to }{r},\overset{\to }{s})\text{.}$$

where $b(\overset{\to }{r},\overset{\to }{s})$ is the real space three point function:

(3.4) $$b(\overset{\to }{r},\overset{\to }{s})\equiv \int_{\overset{\to }{t}}f(\overset{\to }{t})f(\overset{\to }{t}+\overset{\to }{r})f(\overset{\to }{t}+\overset{\to }{s})\text{.}$$

In 2D, the integration leads to:

(3.5) $$\mathrm{RABS}(\overset{\to }{k}q\prime )=\int_{\overset{\to }{r}s\prime }J_{0}(\sqrt{k^{2}r^{2}+q^{2}s^{2}+2\mathrm{kqrs}\cos(\theta_{\mathrm{qk}}-\theta_{\mathrm{sr}})}\;\;\;\;\mathrm{rabs}(\overset{\to }{r}s\prime ),$$

or, equivalently

(3.6) $$\begin{array}{cc} \mathrm{RABS}(k,q,\theta_{\mathrm{qk}})= & \int_{r,s,\theta_{\mathrm{sr}}}J_{0}\left(\sqrt{k^{2}r^{2}+q^{2}s^{2}+2\left(\overset{\to }{k}\cdot \overset{\to }{q}\right)\left(\overset{\to }{r}\cdot \overset{\to }{s}\right)+2(\overset{\to }{k}\times \overset{\to }{q})\cdot (\overset{\to }{r}\times \overset{\to }{s})}\right)\\ \;\;\;\; & \mathrm{rabs}(r,s,\theta_{\mathrm{sr}})\text{.} \end{array}$$

Here $J_{0}$ is the zeroth order Bessel function, and $J_{m}$, as it appears later, is the Bessel function of order *m*. More mathematical details are given in Appendix B. The notation with ′ means that $\overset{\to }{k}q\prime$ depends on the lengths of *k* and *q* and the angle between these two vectors. There is a similar formula in the reverse direction. Notice the last two expressions only depend on shapes (specifically the angles between the vectors). To get the Fourier space RABS of a pattern, one can find its real space rabs and multiply it against the kernel suggested by (3.6). Some geometrical reasoning shows that it is the cross product term in (3.6) which changes sign, when a mirrored image is used: it breaks the mirror symmetry. As noted in the RPS-RDF discussion is the equivalence of the invariants that is noteworthy, for purposes of classification and others. We will discuss more about mirror symmetries in Section 4. That mirroring the image changes the values given by (3.6) is one of the key attributes that makes third order invariants qualitatively more powerful than lower order invariants.

## 4 Gaussian representations for signals: a real space formulation of RABS

The representation of signals by Gaussians has played a prominent role in many scientific endeavors such as structural biology and quantum information science (Grynberg et al., 2010). Unlike wavelets and prolates (Lederman, 2017), such representations do not necessarily form a basis, but have a long history in structural biology since the seminal work of Wilson (Wilson, 1949). Recently have appeared many useful analysis in cryoEM using this latter model, to understand the limiting behavior of FSC curves and the transition to this limit (Singer, Sep 2021), as well as to devise properly constructed correlation functions and construct their asymptotics (Marc Aurèle Gilles and Amit Singer, 2022).

Gaussian signals have also been employed efficaciously in deep learning models (Chen and Ludtke, 2021), and will continue to be part of developing ideas in cryoEM software packages (Bell et al., 2016). Similar to Wilson, we assume that some signal in question can be decomposed into a sum of atoms with identical form factors. This may not be a very good approximation for arbitrary signals, but it is a rich enough representation to show the utility of the third order invariants. Specifically, one can more quickly see the patterning of the third order invariants, when the signal is dominated by punctate points in real space. The Wilson Ansatz leads to the following expressions for the Fourier transform and the radial power spectrum (for example):

(4.1) $$F(\overset{\to }{k})=e^{-\frac{1}{2}k^{2}R^{2}}\overset{N}{\underset{j=1}{\sum }}e^{i\overset{\to }{k}\cdot {\overset{\to }{a}}_{j}},$$

(4.2) RPS(k)=e-k2R2∑j1,j2=1N1SD∫k^eik→·(a→j1-a→j2),

where, as in (2.5), the factor $S_{D}$ is the volume of the *d*-dimensional unit ball. The further evaluation in (4.2) can proceed once the dimension is specified:

(4.3) $$\mathrm{RPS}(k)=e^{-k^{2}R^{2}}\overset{N}{\underset{j_{1},j_{2}=1}{\sum }}\mathrm{Ker}(k|{\overset{\to }{a}}_{j_{1}}-{\overset{\to }{a}}_{j_{2}}|),$$

(4.4) $$\mathrm{Ker}(y)=J_{0}(y),\;\;\;(D=2)$$

(4.5) $$=\frac{\sin y}{y},\;\;\;(D=3)\text{.}$$

There is a nice convenience in that both (4.1) and (4.2) can be decomposed into “self” and “cross” terms. With additional techniques already described, one arrives at, for D = 2:

(4.6) $$\mathrm{RPS}(k)={\mathrm{Ne}}^{-k^{2}R^{2}}+2e^{-k^{2}R^{2}}\overset{N}{\underset{j_{1}<j_{2}=1}{\sum }}J_{0}(k|{\overset{\to }{a}}_{j_{1}}-{\overset{\to }{a}}_{j_{2}}|),$$

(4.7) RDF(t)=Ne-t2/4R24πR2+24πR2∑j1<j2=1Ne-(t-(12))2/4R2I^0(t(12)/2R2).

**Fig. C1:**
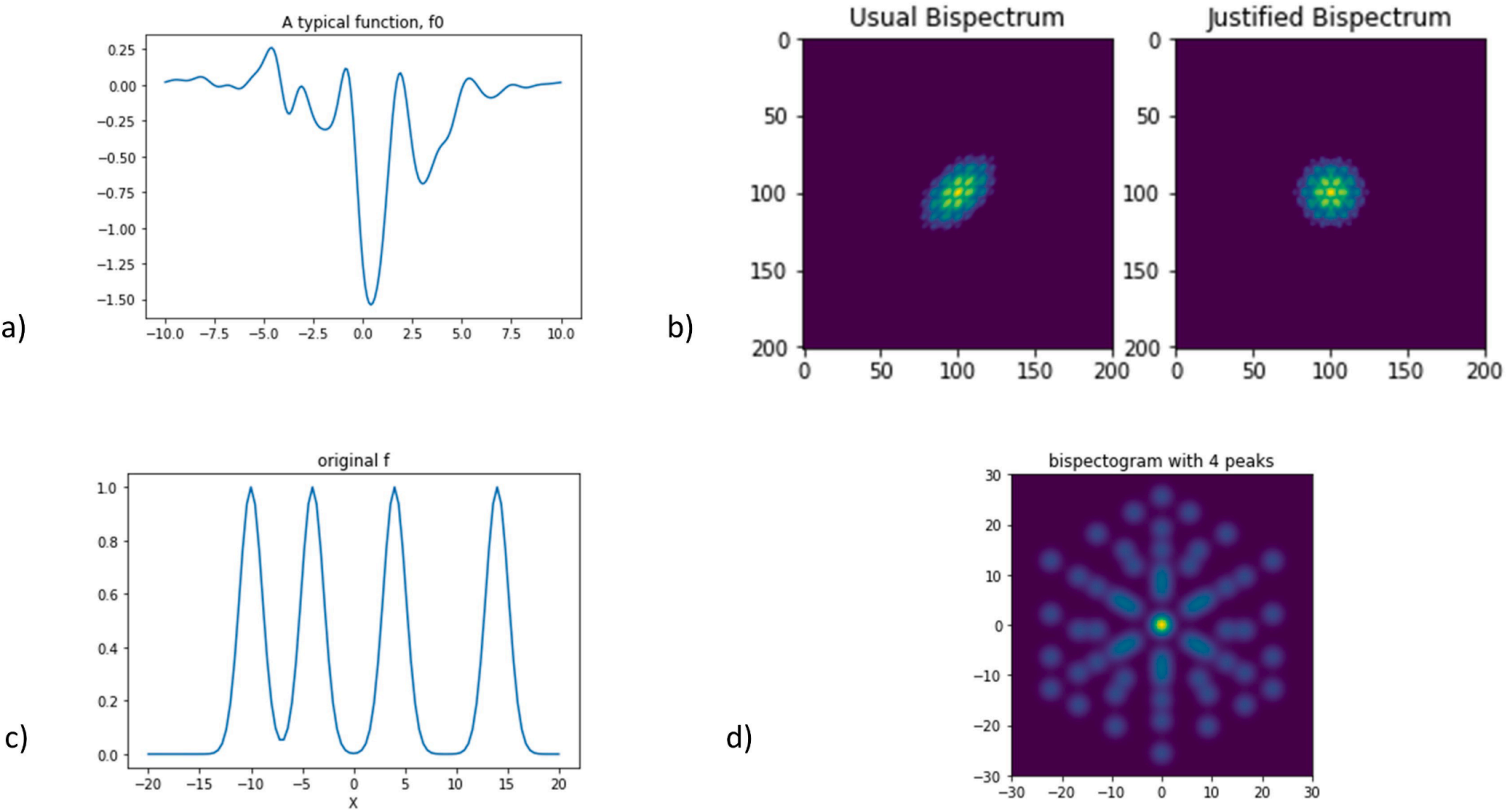
1D bispectrum: Many of the coordinate transformations developed in this manuscript, apply to the 1D bispectrum as well. In a) is a simple nicely windowed and smooth signal. The bispectrum, in frequency space, usually appears (Chua et al., 2008) in the literature as an ellipsoid as in the left side of b). However, with the similar type of coordinate changes introduced here, a 3–2 symmetry emerges, which corresponds to the 6 orderings in size that the lengths of a triangle may have. We call this the justified pattern (in Fourier space) which appears in the right side of b). In c) is drawn a highly punctate 1D signal with 4 peaks. The justified bispectrum is created and inverse Fourier transform can be taken as described in Section 4. Along the 3–2 symmetry lines lie the “skinny triangles” arising from the interference of the same peak twice, whereas the bright spot in the center corresponds to the triple correlations of single peaks. Triangles corresponding to three different peaks lie in the wedge between the 3–2 symmetry lines. Four peaks can form a total of 4 different true triple correlations (combinatorially, this is 4 choose 3). These 4 peaks are shown clearly in the wedges. One can infer the relative positions of the peaks, by the placement of the 4 peaks in the real space wedge. The situation for rabs in arbitrary dimension has all this same facets: i) symmetry hyperplanes corresponding to self terms, and ii) a 6-fold symmetry from which one should choose an asymmetric unit.

Here we use $\overset{\to }{1}2$ to be shorthand for ${\overset{\to }{a}}_{j_{1}}-{\overset{\to }{a}}_{j_{2}}$ etc, and $\left(12)\equiv |\overset{\to }{1}2\right|$. Also I^0(x) is the special function representing the angular average of $e^{-x(1-\cos\theta )}$. The latter is a weak function of *x*, decaying monotonically from 1 to 0, and asymptotically given by $1/\sqrt{2\pi x}$. As seen from inspection from (4.7), RDF(t) will therefore have support for values of *t* that correspond to “interatomic” distances. The first (self) terms in (4.6) and (4.7) are not informative about the relative arrangement of the peaks, and can be essentially neglected as is usually done. In Fig. C1, we show the analogue of this situation for the bispectrum of a one-dimensional signal, where the interatomic distances can be read off from the patterning. The important point is that, just like the speckle imaging work in astronomy, the details of the system (model parameters) can be straightforwardly read out from these patterns.

**A real space formulation of RABS**

In Appendix A, we go through a similar derivation as that which appears above for the two point function, but for the three point function: the real space rabs. However, the main idea in expressing the signal in terms of Gaussians, is that if not so many peaks are necessary to adequately describe the signal in real space, then it is more effective to describe RABS in real space. Moreover there is a big advantage that there will not be aliasing in the real space version. There is much more discussion in Appendix C, and in Fig. C1, where we show that the “skewing” of the bispectrum can be justified by the same choice of linear combinations of variables.

The final expression for the rotationally averaged correlation function is given by:

(4.8) rabs(v,w,θwv)=12πR23∑j1,j2,j3=1nG(1)(v-av,R314)G(1)(w-aw,R314)I^0(Y)e-(vav+waw-Y)3R2,

where $G^{\left(1\right)}(x,S)=\frac{1}{\sqrt{2\pi S^{2}}}\exp^{-\frac{x^{2}}{2S^{2}}}$, is the properly normalized 1D Gaussian. Also

(4.9) $$Y=\sqrt{v^{2}a_{v}^{2}+w^{2}a_{w}^{2}+2{\mathrm{wva}}_{v}a_{w}\cos(\theta_{a_{w}a_{v}}-\theta_{\mathrm{wv}})}/(\sqrt{3}R^{2}),$$

and

(4.10) a→v=33413∑k=13a→jk-a→j^1“drop”,

(4.11) a→w=3142(a→j^2-a→j^3)“shortside”.

What we have termed “the drop” is a rescaled version of what is called the triangle median, and opposing the shortest side.

**Fig. 2:**
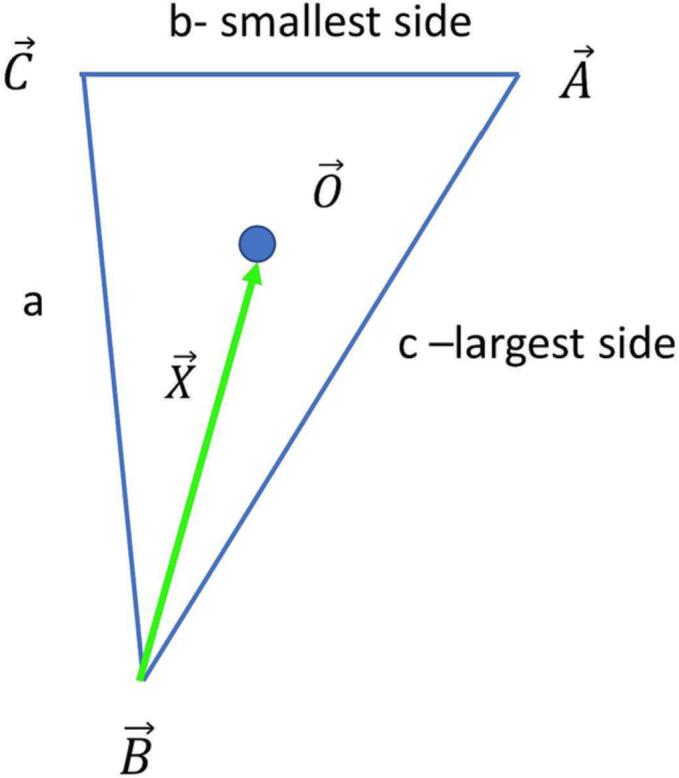
An explication of convenient coordinates for triangles, stemming originally from a correlation analysis. Sides are denoted by a,b, and *c*, and are opposite the corresponding vertices, $\overset{\to }{A},\overset{\to }{B}$, and $\overset{\to }{C}$. The shortest and longest sides are termed b and c respectively. The median $\overset{\to }{O}$ of the triangle is constructed as well as the vector from $\overset{\to }{B}$ to $\overset{\to }{O}$, which we term “the drop”, $\overset{\to }{X}$. The three coordinates describing the triangle are given by the lengths, X, b and by the angle between $\overset{\to }{A}-\overset{\to }{C}$ and $\overset{\to }{X}$. This angle is always less than or equal to π/2 in magnitude. Mirroring the triangle can be represented by changing the sign of this last angle.

As mentioned already I^0(y)≡e-yI0(y) is a weak function of *y* with an algebraically decaying tail. So the more important dependence of the expression (4.8) on the angle is given by the neighboring term in (4.8) given by $e^{-(\frac{v\;a_{v}+w\;a_{w}-Y)}{3R^{2}}}$, which is also a Gaussian in the angle, in the small *R* limit. In the expressions (4.10) and (4.11), the vector ${\overset{\to }{a}}_{w}$ represents the shortest side of the triangle given by the three points $j_{1},\;j_{2},\;j_{3}$. The direction is toward the vertex which belongs to the longest leg. The vector a→j^1, represents the vertex opposite the shortest leg of the triangle. The scenario is shown in Fig. 2. The expression in the radical in (4.9) can be reorganized in the same way that was done in (3.6).

(4.12) $$Y=\sqrt{v^{2}a_{v}^{2}+w^{2}a_{w}^{2}+2(\overset{\to }{v}\cdot \overset{\to }{w})\;({\overset{\to }{a}}_{v}\cdot {\overset{\to }{a}}_{w})+2(\overset{\to }{v}\times \overset{\to }{w})\cdot ({\overset{\to }{a}}_{v}\times {\overset{\to }{a}}_{w})}\text{.}$$

**Asymmetric Unit of real space, rabs, and the discernment of mirrors**

Given the triple of points $j_{1},\;j_{2},\;j_{3}$, there is always an essentially unique definition, then to describe the triangle using ${\overset{\to }{a}}_{v}$ and ${\overset{\to }{a}}_{w}$: the situation does not depend on the order in which $j_{1},\;j_{2}$, and $j_{3}$ are presented. Moreover, given these definitions there is a an asymmetric unit as discussed in Appendix D. Using the definition for θ as the angle between ${\overset{\to }{a}}_{v}$ and ${\overset{\to }{a}}_{w}$, with θ>0 meaning that ${\overset{\to }{a}}_{v}$ leads ${\overset{\to }{a}}_{w}$ in the clockwise sense. It is easy to show -π/2⩽θ⩽π/2 and moreover $a_{w}⩽a_{v}(\sqrt{3+\cos^{2}\theta }-\cos\theta )\frac{1}{\sqrt{6}}$, as seen in Fig. 2 and derived in Appendix D.

## 5 Results

**Fig. 3:**
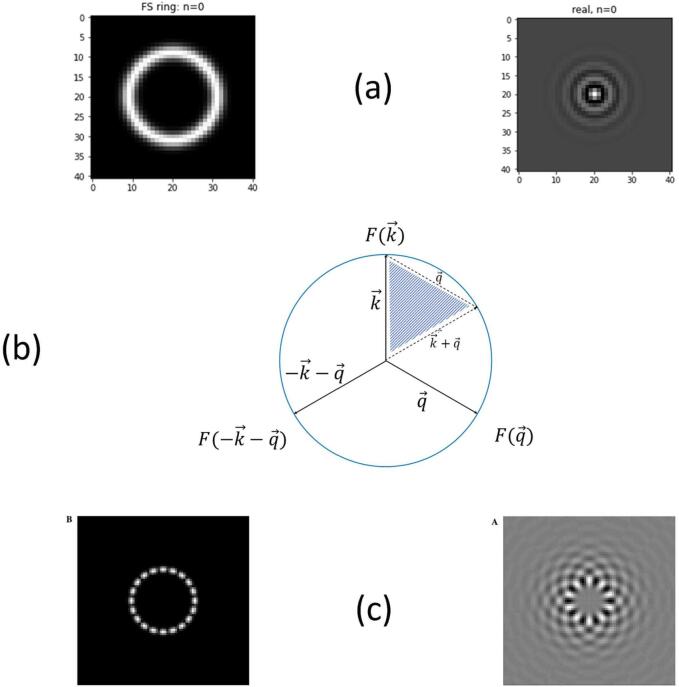
Example of compact Fourier Space pattern and its third order invariant. In (a) on the left is a Fourier space pattern in 2D which has a rotational symmetry and is concentrated on a ring at density, 1/R. The real space pattern is on the right. In Fourier space, the third order correlation function is built from three vectors of the form shown in (b). There is a unique shape such that all three wavevectors: $\overset{\to }{k},\;\overset{\to }{q},\;-\overset{\to }{k}-\overset{\to }{q}$ lie on the circle as shown. The vectors, $\overset{\to }{q}$, and $\overset{\to }{k}+\overset{\to }{q}$ are shown a second time bordering the shaded area in panel (b). Using this idea, it is easy to construct a function such as described in Fourier (left) and real (right) spaces as shown in panel (c), that have everywhere vanishing RABS as given in Eq. 5.2.

We give examples of simple patterns allowing for evaluations of the third order correlations in either real or reciprocal spaces. If the data is not highly compact in real space, it is typically more efficient to evaluate the third order invariants starting from Fourier space. We will discuss this approach at length in later work using our cryoEM software EMAN2. A simple function which is compact in Fourier space and has a natural looking pattern in real space is given by a function which has only support over a circle in Fourier space. For example in D = 2, the function given by

(5.1) $$f(\overset{\to }{r})=J_{0}(r/R),$$

has a Fourier transform which is concentrated on a ring at Fourier radius, 1/R. In this case, the only triangles that can be constructed in Fourier space, whose wavevectors sum to zero (necessary by translational invariance and discussed below Eq. 3.1), are given by equilateral triangles, as shown in Fig. 3. In that case the geometry is therefore fixed by a single type of triangle, so that the support of the Fourier space RABS is simply a single point, corresponding to an equilateral triangle of side length $\sqrt{3}/R$ (in Fourier space). Indeed all of the individual Fourier harmonics (Baldwin and Penczek, 2005) have RABS, which have support at either one point or have RABS which are identically zero. For example, the function

(5.2) $$\cos(2m\theta_{r})J_{2m}(r/R),$$

which is depicted on the right side of Fig. 3c (the Fourier amplitude is on the left side of Fig. 3c), can be shown to have a vanishing RABS, for all integer, *m* (in the Figure m=10). This shows immediately that 3rd order invariants cannot act perfectly as classifiers.

**Fig. 4:**
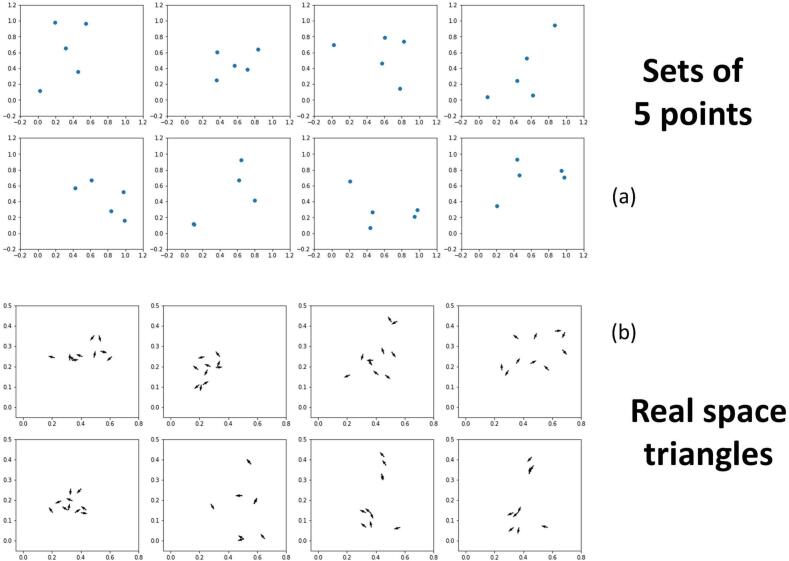
Examples of patterns compact in (a) real space (5 points) and their (b) third order invariants as given by Eq. (4.8). The real space invariants are expressed by writing down for each of the 10 triangles formed by the 5 points, the 1. short side, 2. the drop (as discussed in Fig. 3 and the angle between these two vectors. Short side and drop are given by the two axes shown, whereas the angle is represented by the direction of the arrow on top of the point. Each pattern of invariants are very different except for the pattern of invariants of the last two of the (b) subpanel. The original real space set of points are shown in the last two plots of panel (a); these patterns are clearly seen to be mirrors. In the invariants, this corresponds to mirroring the vectors across the x-axis.

This manuscript is motivated by the idea that the image invariants may be formulated in the space where the original function is most compact. In the case of sums of Gaussians, which has become a repeatedly useful representation for data in cryo EM for a multitude of reasons, we arrived at the expressions derived in Section 4, where there are peaks in the real space RABS representation. If, in addition, the width of the original Gaussians is exceedingly small, the functional behavior of the expression (4.8) in terms of angle is also singular and Gaussian. Indeed the expression (4.9) describes a representation that recreates the triangles of the original signal as in (3.2). Examining Fig. 4, the 5 points in an original signal in (a) create 10 triangles in (b) as given by Eq. 4.8). These are the 10 points shown there with a representation where the angles ($\theta_{\mathrm{wv}}$) are shown by a vector, so that the data can be shown conveniently in 2D (and not 3D). All of the patterns of third order invariants look completely unlike one another, except for the third order invariants representing the last two patterns, which are mirrors. The invariants for the mirrors can be transformed into one another by mirroring the vector across the x-axis, which sends the angle to its negative as described in Fig. 2. That mirrors can be distinguished by third order quantities is one of the features that make them attractive as a classifier, and why we have gone over the aspect in such detail.

**Fig. 5:**
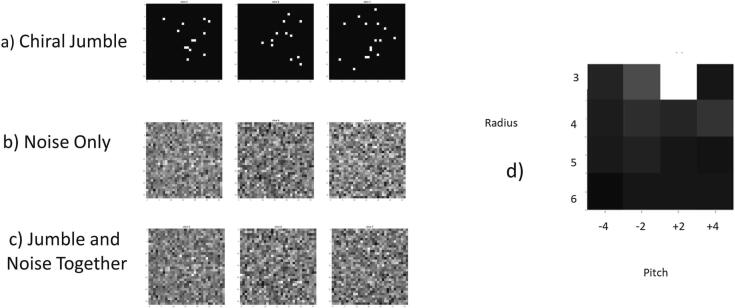
We create a jumble of chiral “propellers” as described in the text. A single plane of the volume of a combined pattern of 100 of such fragments is shown on the first row of a). Gaussian noise is added to it as shown in b), with the resultant shown in c). The SNR can be estimated as 6%. Four point correlations are then sought using the same style of sampling but with varying radius and (signed) pitch with a strong resonance at the correct value. A mirror operation in 3D, changes the chirality of an object and would take a positive pitch object to negative pitch, but the pitch is clearly distinguished as shown in d). This is precursor to study of the trispectrum.

Looking forward, we note that third order correlations are able to distinguish mirrors and intrinsically 2D textures, whereas it is necessary to go to a 4 point function to study 3D textures. Toward that end, in Fig. C1 we give briefly an example of a four point function that is a 4 point sampling of a single turn of a helix, and the efficacy of identifying a mix of such objects in large noise within a small volume. Four points are assigned a unit value at a radial distance *R* from the z-axis and also at π/2 from each other, as one progresses one unit of “pitch” along the *z* axis, to create the simplest complete turn of a helix that can be imagined. One hundred of such entities are inserted into a $32^{3}$ array, at random positions, and also at one of the 24 directions corresponding to cubic symmetry. The situation is summarized in Fig. 5. Gaussian noise (Fig. 5b) at amplitude level 0.45 is now added to each voxel as shown, resulting in the third row, where any systematic amount of signal is not discernible to the eye. There is a strong resonance of a high quality factor (peak at correct radius and pitch six times that of the other peaks). The SNR for the original signal can be estimated as 4x100 (that is, the number of points in the propeller times the number of propellers) divided by the number of voxels divided by the strength of the noise, which gives a value of 6%. This is empirically the highest noise level at which we can still see a good resonance.

## 6 Discussion

We have given formulae relating motion invariants formulated in either real or Fourier spaces. We have given examples of such motion invariants, depending on which space forms the most compact representation. Ultimately, motion invariants of signals can play a prominent role in classification of signals in cryoEM, and is a core part of the (cryoEM) EMAN2 classification scheme. So it is imperative how many invariants there are, and how they are related.

For an arbitrary signal, it is generally numerically advantageous to formulate the motion invariants in Fourier space, because one less volume integration needs to be performed (the invariants are a real space convolution). We saw that it was easy to construct signals that have dense support in real space, but have vanishing third order correlations, so that the third order motion invariants can never act as a perfect classifier. In general, one can argue that the third order represents an interference pattern of Fourier harmonic modes (Baldwin and Penczek, 2005), and we expect, but cannot prove, that the original signal can be reconstructed, in the typical case, from the motion invariants.

In the real space, we showed examples where a signal was comprised of Gaussians of the same strength and shape, but otheriwise different centers. For a highly punctate signal, the real space third order correlation (RABS) simply reproduces, all of the real space triangles that appear on the pattern. Indeed, this is a numerically very efficient way to characterize the image. It seems likely that in general one should be able to recreate the placement of *N* points on the plane by knowledge of the $\frac{N(N-1)(N-2)}{6}$ triangles that they form.

The problem is reminiscent of multidimensional scaling, where cities can be placed on the plane by knowing the intercity distances. Multidimensional scaling algorithms can accurately estimate city positions from inter-city distances, up to an overall mirroring. The inter-city distances contain information that is essentially equivalent to the radial power spectrum. However, a more detailed representation of the signal can be obtained by annotating all the possible triangles formed from the cities. This triangle representation is related to the information contained in the rabs function. Such an annotation is, in contrast, able to distinguish the overall mirror of the pattern, for example.

The formulation of triangles that we reproduce here should be compared with so-called Bookstein (Dryden and Mardia, 2016) coordinates, which represent triangles as two dimensional points, because the scale of the triangles are considered to be set. We consider that the representation here is more symmetric.

## Declaration of Competing Interest

The authors declare that they have no known competing financial interests or personal relationships that could have appeared to influence the work reported in this paper.

## Data Availability

No data was used for the research described in the article.

## Appendix A Relation between RPS and RDF

In this appendix, we show the equivalence of the rotational invariants formed by the two point functions: RPS, which is formulated in Fourier space, and RDF in real space. These are more of the technical details than what appears in the main body of the text in Section 2. The construction for the transcription for the higher order correlation functions take place with a similar mechanism. Consider a real 2D signal, *f*, and its Fourier transform, *F*, as well as its squared modulus:

(A.1) $$|F(\overset{\to }{k}){|}^{2}=\int_{\overset{\to }{r},\overset{\to }{s}}f(\overset{\to }{r})f(\overset{\to }{s})e^{i\overset{\to }{k}\cdot (\overset{\to }{r}-\overset{\to }{s})},$$

(A.2) $$\;=\int_{\overset{\to }{t},\overset{\to }{s}}f(\overset{\to }{s}+\overset{\to }{t})f(\overset{\to }{s})e^{i\overset{\to }{k}\cdot \overset{\to }{t}}\text{.}$$

In going from (A.1) to (A.2), we have used $\overset{\to }{r}=\overset{\to }{s}+\overset{\to }{t}$. Notice that because the integration kernel depends on the product $\overset{\to }{k}\cdot \overset{\to }{t}$ (the transform is “axial”), that an angular average of the kernel over k^ has the same effect as an angular average over t^ inside the integral. We use this idea throughout these derivations. We can use this idea to calculate the rotational average of the power spectrum, which is mainly called the Radial Power spectrum (RPS):

(A.3) RPS(k)≡1SD∫k^|F(k→)|2,

(A.4) =∫t1SD∫k^eik→·t→∫s→f(s→)∫t^f(s→+t→),

(A.5) =SD∫t1SD∫k^eik→·t→RDF(t),

(A.6) $$\;=S_{D}\int_{0}^{\infty }dt\;\;t^{D-1}\;\;\mathrm{Ker}(\mathrm{kt})\mathrm{RDF}(t),$$

(A.7) Ker(kt)≡1SD∫k^eik→·t→,

where $S_{D}$ is the surface area of the D dimensional ball. For example, $S_{2}=2\pi ,\;\;\;S_{3}=4\pi ,S_{4}=2\pi^{2}$, and the general expression can be found many places. Also RDF(t) in (A.5), the radial distribution function, is given by:

(A.8) RDF(t)≡1SD∫s→,t^f(s→)f(s→+t→).

In 2D, using $\mathrm{Ker}(\mathrm{kt})=J_{0}(\mathrm{kt})$, the expression (A.6) now becomes

(A.9) $$\mathrm{RPS}(k)=2\pi \int_{0}^{\infty }dt\;t\;J_{0}(\mathrm{kt})\;\;\mathrm{RDF}(t),$$

where $J_{0}$ is the zeroth order Bessel function of the first kind.

The reciprocal can be found in a similar manner. Substituting for the Fourier transforms in (A.8), via:

(A.10) RDF(t)=1SD∫k→∫l→F(k→)F(-l→)12π2D∫s→,t^e-ik→·s→eil→·(s→+t→),

(A.11) =1SD∫k→F(k→)F(-k→)12πD∫t^eik→·t→,

(A.12) =12πD∫k∫t^eik→·t→SD∫k^F(k→)F(-k→),

(A.13) $$\;=S_{D}{\left(\frac{1}{2\pi }\right)}^{D}\int_{0}^{\infty }dk\;\;k^{D-1}\;\;\mathrm{Ker}(\mathrm{kt})\;\;\mathrm{RPS}(k)\text{.}$$

In 2D, again using $\mathrm{Ker}(\mathrm{kt})=J_{0}(\mathrm{kt})$, the last expression becomes:

(A.14) $$\mathrm{RDF}(t)=\frac{1}{2\pi }\int_{0}^{\infty }dk\;k\;J_{0}(\mathrm{kt})\;\;\mathrm{RPS}(k)\text{.}$$

The transcriptions given by (A.9) and (A.14) are well known. They are consistent with the orthogonality condition

(A.15) $$\int_{0}^{\infty }dk\;k\;J_{0}(\mathrm{kt})J_{0}(\mathrm{ks})=\frac{1}{s}\delta (t-s)\text{.}$$

More common are the orthogonality relationships between Bessel functions using Bessel zeros and finite intervals, but (A.15) also exists (Watson, 1944) as a formal result

(A.16) $$\int_{0}^{\infty }dk\;k\;J_{m}(\mathrm{kt})J_{m}(\mathrm{ks})=\frac{1}{s}\delta (t-s)\text{.}$$

The general orthogonality condition can be written as

(A.17) $$\frac{S_{D}^{2}}{{\left(2\pi \right)}^{D}}\int_{0}^{\infty }dk\;k^{D-1}\;\mathrm{Ker}(\mathrm{kt})\;\mathrm{Ker}(\mathrm{ks})=\frac{1}{t^{D-1}}\delta (t-s)\text{.}$$

In each dimension, *D*, we simply needed to evaluate the average of the phase over the unit sphere to get Ker, and then use (A.6), (A.13), and (A.17) to get expressions for the RPS, RDF, and orthogonality relationship. For D=3, we have

(A.18) $${\mathrm{Ker}}_{D=3}(x)=\frac{\sin x}{x},$$

(A.19) $$\mathrm{RPS}(k)=4\pi \int_{0}^{\infty }\;dt\;t^{2}\;\frac{\sin\mathrm{kt}}{\mathrm{kt}}\;\;\mathrm{RDF}(t),$$

(A.20) $$\mathrm{RDF}(t)=\frac{1}{2\pi^{2}}\int_{0}^{\infty }dk\;k^{2}\;\frac{\sin\mathrm{kt}}{\mathrm{kt}}\;\;\mathrm{RPS}(k)\text{.}$$

For D = 4, we simply write down the kernel, by which all of the other expressions follow.

(A.21) $${\mathrm{Ker}}_{D=4}(x)=J_{0}(x)+J_{2}(x)\text{.}$$

This completes the derivations that appear in Section 2, between the simplest set of motion invariants: the RPS and RDF.

## Appendix B Three point correlation function (3PCF): Fourier space representation and transformation to real space (rabs)

In this appendix, we give more details to the derivations in Section 3, relating three point motion invariants between real (rabs) and Fourier space (RABS). The RABS is easier to develop computationally in Fourier space generally, however we find it easiest to describe intuitively the meaning of the rotationally averaged three point function as the resonance of the original signal with triangles of a given shape.

To proceed, we formulate the 3PCF in Fourier Space:

(B.1) $$B(\overset{\to }{k},\overset{\to }{q})\equiv F(\overset{\to }{k})F(\overset{\to }{q})F(-\overset{\to }{k}-\overset{\to }{q}),$$

(B.2) $$\;=\int_{\overset{\to }{r},\overset{\to }{s},\overset{\to }{t}}f(\overset{\to }{t}+\overset{\to }{r})f(\overset{\to }{t}+\overset{\to }{s})f(\overset{\to }{t})e^{i\overset{\to }{k}\cdot \overset{\to }{r}}e^{i\overset{\to }{q}\cdot \overset{\to }{s}},$$

(B.3) $$\;=\int_{\overset{\to }{r},\overset{\to }{s}}e^{i\overset{\to }{k}\cdot \overset{\to }{r}}e^{i\overset{\to }{q}\cdot \overset{\to }{s}}\;\;b(\overset{\to }{r},\overset{\to }{s})\text{.}$$

where $b(\overset{\to }{r},\overset{\to }{s})$ is the real space three point function (bispectrum):

(B.4) $$b(\overset{\to }{r},\overset{\to }{s})\equiv \int_{\overset{\to }{t}}f(\overset{\to }{t})f(\overset{\to }{t}+\overset{\to }{r})f(\overset{\to }{t}+\overset{\to }{s})\text{.}$$

To make the rest of the argument simpler, we introduce 4-vectors

(B.5) $$\overset{\to }{r}s=(r1,r2,s1,s2),$$

(B.6) $$\overset{\to }{k}q=(k1,k2,q1,q2),$$

where $\overset{\to }{r}\equiv (r1,r2)$, etc. Then Eq (B.3) may be rewritten:

(B.7) $$B(\overset{\to }{k}q)=\int_{\overset{\to }{r}s}e^{i\overset{\to }{k}q\cdot \overset{\to }{r}s}\;\;b(\overset{\to }{r}s),$$

Here $\overset{\to }{k}q\cdot \overset{\to }{r}s=k1\cdot r1+k2\cdot r2+q1\cdot s1+q2\cdot s2$is a useful shorthand.

The idea now is that if one does a global rotation of $\overset{\to }{k}q$ while holding the angle between *k* and *q* constant (the shape of the Fourier space triangle), this leads to

(B.8) RABS(k→q′)=∫k^B(k→q),

(B.9) =∫r→s∫k^eik→q·r→sb(r→s),

(B.10) =∫r→s′∫k^eik→q·r→s∫r^b(r→s),

(B.11) =∫r→s′∫k^eik→q·r→srabs(r→s′).

The notation with ′ means that $\overset{\to }{k}q\prime$ depends on the lengths of *k* and *q* and the angle between these two vectors. Notice that, requiring that there be translational invariance, requires that the correlation function in Fourier space be built from Fourier vectors, whose vector sum is zero as can be noticed from (3.1). This is true for arbitrary dimension and correlation function order. In 2D, the integration leads to:

(B.12) $$\mathrm{RABS}(\overset{\to }{k}q\prime )=\int_{\overset{\to }{r}s\prime }J_{0}(\sqrt{k^{2}r^{2}+q^{2}s^{2}+2\mathrm{kqrs}\cos(\theta_{\mathrm{qk}}-\theta_{\mathrm{sr}})}\;\;\;\;\mathrm{rabs}(\overset{\to }{r}s\prime ),$$

or, equivalently

(B.13) $$\begin{array}{cc} \mathrm{RABS}(k,q,\theta_{\mathrm{qk}})= & \int_{r,s,\theta_{\mathrm{sr}}}J_{0}\left(\sqrt{k^{2}r^{2}+q^{2}s^{2}+2\left(\overset{\to }{k}\cdot \overset{\to }{q}\right)\left(\overset{\to }{r}\cdot \overset{\to }{s}\right)+2(\overset{\to }{k}\times \overset{\to }{q})\cdot (\overset{\to }{r}\times \overset{\to }{s})}\right)\\ \;\;\;\; & \mathrm{rabs}(r,s,\theta_{\mathrm{sr}})\text{.} \end{array}$$

There is a similar formula in the reverse direction, taking real space rabs to its Fourier version. Notice the last two expressions only depend on shapes (specifically the angles between the vectors). To get the Fourier space RABS of a real space pattern, one can find its real space rabs and multiply it against the kernel suggested by (B.13). Some geometrical reasoning shows that it is the cross product term in (B.13) which changes sign, when a mirrored image is used: it breaks the mirror symmetry. We discuss more about mirror symmetries in Section 4, using the real space formulation of invariants.

## Appendix C Gaussian Model of Signal for Third Order Invariants

We begin with (3.4)

(C.1) $$b(\overset{\to }{r},\overset{\to }{s})\equiv \int_{\overset{\to }{t}}f(\overset{\to }{t})f(\overset{\to }{t}+\overset{\to }{r})f(\overset{\to }{t}+\overset{\to }{s})\text{.}$$

and the expression in real space for the signal consistent with (4.1) which is

(C.2) $$f(\overset{\to }{t})=\overset{N}{\underset{j=1}{\sum }}G^{\left(2\right)}(\overset{\to }{t}-{\overset{\to }{a}}_{j},R),$$

where $G^{\left(2\right)}$ is the properly normalized 2D Gaussian consistent with (C.1). Most explicitly $G^{\left(2\right)}(\overset{\to }{r},R)\equiv \frac{1}{2\pi R^{2}}e^{-\frac{r^{2}}{2R^{2}}}$. To perform the *t* integration, it is convenient to complete terms in the resulting exponential in (C.1) in the most symmetric fashion possible:

(C.3) $$\begin{array}{cc} {\left(\overset{\to }{t}-{\overset{\to }{a}}_{j_{1}}\right)}^{2}+{\left(\overset{\to }{t}+\overset{\to }{r}-{\overset{\to }{a}}_{j_{2}}\right)}^{2}+{\left(\overset{\to }{t}+\overset{\to }{s}-{\overset{\to }{a}}_{j_{3}}\right)}^{2} & =\\ & 3{\left(\overset{\to }{t}-\frac{3\overset{\to }{O}-\overset{\to }{r}-\overset{\to }{s}}{3}\right)}^{2}+\frac{2}{3}(r^{2}-\overset{\to }{r}\cdot \overset{\to }{s}+s^{2})-2{\overset{\to }{a}}_{j_{2}}\prime \cdot \overset{\to }{r}-2{\overset{\to }{a}}_{j_{3}}\prime \cdot \overset{\to }{s}+\overset{3}{\underset{p=1}{\sum }}{\left(a_{j_{3}}\prime \right)}^{2}\text{.} \end{array}$$

where

(C.4) $$\overset{\to }{O}\equiv \frac{1}{3}\overset{3}{\underset{k=1}{\sum }}{\overset{\to }{a}}_{j_{k}},$$

is the median of the three points and

(C.5) $${\overset{\to }{a}}_{j_{k}}\prime \equiv {\overset{\to }{a}}_{j_{k}}-\overset{\to }{O}\text{.}$$

We wish to further transform to eliminate cross terms in (C.1). One notes

(C.6) $$r^{2}-\overset{\to }{r}\cdot \overset{\to }{s}+s^{2}=\frac{1}{4}{\left(\overset{\to }{r}+\overset{\to }{s}\right)}^{2}+\frac{3}{4}{\left(\overset{\to }{r}-\overset{\to }{s}\right)}^{2}\text{.}$$

This leads one to be able to write (C.3) as

(C.7) $$3{\left(\overset{\to }{t}-\frac{3\overset{\to }{O}-\overset{\to }{r}-\overset{\to }{s}}{3}\right)}^{2}+\frac{1}{6}{\left(\overset{\to }{r}+\overset{\to }{s}+3{\overset{\to }{a}}_{j_{1}}\prime \right)}^{2}+\frac{1}{2}{\left(\overset{\to }{r}-\overset{\to }{s}-({\overset{\to }{a}}_{j_{2}}\prime -{\overset{\to }{a}}_{j_{3}}\prime )\right)}^{2}$$

One makes a final area preserving change of coordinates

(C.8) $$3^{\frac{1}{4}}\;\;\overset{\to }{v}\equiv \frac{1}{\sqrt{2}}(\overset{\to }{r}+\overset{\to }{s}),$$

(C.9) $$3^{-\frac{1}{4}}\;\;\overset{\to }{w}\equiv \frac{1}{\sqrt{2}}(\overset{\to }{r}-\overset{\to }{s})\text{.}$$

so that the variances of the remaining Gaussians are identical. These types of transformations yield, the types of patterns in Fig. C1.

So finally (C.3) has become

(C.10) $$\begin{array}{cc} {\left(\overset{\to }{t}-{\overset{\to }{a}}_{j_{1}}\right)}^{2}+{\left(\overset{\to }{t}+\overset{\to }{r}-{\overset{\to }{a}}_{j_{2}}\right)}^{2}+{\left(\overset{\to }{t}+\overset{\to }{s}-{\overset{\to }{a}}_{j_{3}}\right)}^{2} & =\\ & 3{\left(\overset{\to }{t}-\frac{3\overset{\to }{O}-\overset{\to }{r}-\overset{\to }{s}}{3}\right)}^{2}+\frac{1}{\sqrt{3}}{\left(\overset{\to }{v}-{\overset{\to }{a}}_{v}\right)}^{2}+\frac{1}{\sqrt{3}}{\left(\overset{\to }{w}-{\overset{\to }{a}}_{w}\right)}^{2}, \end{array}$$

(C.11) $${\overset{\to }{a}}_{v}=\frac{3^{-\frac{1}{4}}}{\sqrt{2}}\left(-3{\overset{\to }{a}}_{j_{1}},\prime \right)$$

(C.12) $${\overset{\to }{a}}_{w}=\frac{3^{\frac{1}{4}}}{\sqrt{2}}({\overset{\to }{a}}_{j_{2}}-{\overset{\to }{a}}_{j_{3}})\text{.}$$

Now that we have completed the square, the $\overset{\to }{t}$ convolution in (C.1) can be performed:

(C.13) $$\overset{¯}{b}(\overset{\to }{v},\overset{\to }{w})=\overset{n}{\underset{j_{1},j_{2},j_{3}=1}{\sum }}G^{\left(2\right)}(\overset{\to }{v}-{\overset{\to }{a}}_{v},R\;3^{\frac{1}{4}})\;\;G^{\left(2\right)}(\overset{\to }{w}-{\overset{\to }{a}}_{w},R,3^{\frac{1}{4}})\text{.}$$

Finally we would like to push this in a form that is amenable to the final overall angular integration:

(C.14) $$\frac{1}{2}{\left(\overset{\to }{v}-{\overset{\to }{a}}_{v}\right)}^{2}+\frac{1}{2}{\left(\overset{\to }{w}-{\overset{\to }{a}}_{w}\right)}^{2}=\frac{1}{2}{\left(v-a_{v}\right)}^{2}+\frac{1}{2}{\left(w-a_{w}\right)}^{2}+({\mathrm{va}}_{v}+{\mathrm{wa}}_{w}-\overset{\to }{v}\cdot {\overset{\to }{a}}_{v}-\overset{\to }{w}\cdot {\overset{\to }{a}}_{w})$$

We are rewriting the symbols so that it is easy to see that in the limit of very punctate data (R→0, tight peaks), the variables take on values corresponding to the same triangles that appear in the original 2D pattern.

So after the angular average of (C.13), while keeping the shape of the triangle constant

(C.15) $$\mathrm{rabs}(v,w,\theta_{\mathrm{wv}})=\frac{1}{2\pi R^{2}\sqrt{3}}\overset{n}{\underset{j_{1},j_{2},j_{3}=1}{\sum }}G^{\left(1\right)}(v-a_{v},R\;3^{\frac{1}{4}})\;\;G^{\left(1\right)}(w-a_{w},R\;3^{\frac{1}{4}})e^{-\frac{v\;a_{v}+w\;a_{w}}{\sqrt{3}R^{2}}}\frac{1}{2\pi }\int_{\theta_{v}}e^{-(\overset{\to }{v}\cdot {\overset{\to }{a}}_{v}+\overset{\to }{w}\cdot {\overset{\to }{a}}_{w})/\sqrt{3}R^{2}},$$

where $G^{\left(1\right)}(x,S)=\frac{1}{\sqrt{2\pi S^{2}}}\exp^{-\frac{x^{2}}{2S^{2}}}$ is the properly normalized 1D Gaussian.

The last integral is performed, while the angle between $\overset{\to }{v}$and $\overset{\to }{w}$ is constant. The answer to the last integral is:

(C.16) $$I_{0}\left(Y\right),\;\text{with}\;Y\equiv \sqrt{v^{2}a_{v}^{2}+w^{2}a_{w}^{2}+2{\mathrm{wva}}_{v}a_{w}\cos\left(\theta_{a_{w}a_{v}}-\theta_{\mathrm{wv}}\right)}/\left(\sqrt{3}R^{2}\right)$$

Recall that I^0(y)≡e-yI0(y) is a weak function of *y* with an algebraically decaying tail. So

(C.17) rabs(v,w,θwv)=12πR23∑j1,j2,j3=1nG(1)(v-av,R314)G(1)(w-aw,R314)I^0(Y)e-(vav+waw-Y)3R2.

**The singular limit** R→0 **.**

From the first two terms, clearly the singular limit constrains $v=a_{v}$ as well as $w=a_{w}$. However the term in the last exponential attains its maximum when the angle $\theta_{\mathrm{vw}}$ matches the angle that rotates $a_{w}$ along $a_{v}$ at which this term vanishes. So, using asymptotics, one may also write this as a Gaussian in the angle.

The upshot, is that if the initial signal is highly punctate, then the bispectrum as expressed in real space also is highly punctate. And it simply represents the patterns of all possible triangles that can be drawn, where any possible triple of points that might comprise a triangle are represented by a single point in the asymmetric unit of the real space RABS.

This is not so easy to visualize as it takes place in a 3D space. In Fig. 4, we used a convenient representation to express the situation in a 2D plane, by using a phase to represent an interior angle of the triangle.

## Appendix D Asymmetric Unit Real Space Rabs

In order to plot the real space version of RABS suggested in Section 4, one must carefully consider the domain of the variables representing triangles, which is not simple, in general. Starting from the Fig. 2, we look for a constraint between the short side length, the length of the drop, and the angle, θ, between them. The angle is termed positive if $\overset{\to }{A}-\overset{\to }{C}$ leads $\overset{\to }{X}$ in the clockwise sense as is the case in the example shown in the figure. The mirrored triangle would have a negative θ. One defines:

(D.1) $$3\overset{\to }{X}=\overset{\to }{A}+\overset{\to }{C}-2\overset{\to }{B}$$

leading to

(D.2) $$9X^{2}=a^{2}+c^{2}+2\left(\overset{\to }{A}-\overset{\to }{B}\right)\cdot \left(\overset{\to }{C}-\overset{\to }{B}\right)\;.$$

But one can use the law of cosines to reduce this to

(D.3) $$9X^{2}=2a^{2}+2c^{2}-b^{2}\;.$$

On the other hand,

(D.4) $$3\mathrm{Xb}\cos\theta =3\overset{\to }{X}\cdot (\overset{\to }{A}-\overset{\to }{C})=|\overset{\to }{A}-\overset{\to }{B}{|}^{2}-|\overset{\to }{C}-\overset{\to }{B}{|}^{2}=c^{2}-a^{2}$$

Recall from the figure: c⩾a⩾b So

(D.5) $$9X^{2}-6\mathrm{Xb}\cos\theta =4a^{2}-b^{2}⩾3b^{2}$$

So

(D.6) $${\left(b+X\cos\theta \right)}^{2}⩽3X^{2}+\cos\theta^{2}X^{2}$$

And finally:

(D.7) $$b⩽X(\sqrt{3+\cos^{2}\theta }-\cos\theta )$$

Every triangle has a unique presentation in the region given by $\left|\theta |⩽\pi /2,b⩽X(\sqrt{3+\cos^{2}\theta }-\cos\theta \right)$ Recall that *b* was singled out as the smallest of the three sides, and the definition of θ was such that it is less in magnitude to π/2.
